# Physical Disability, Service Coordination, & Outcomes in Publicly-Funded Long-Term Services & Supports

**DOI:** 10.1093/geroni/igaf122.013

**Published:** 2025-12-31

**Authors:** Romil Parikh, Dana Urbanski, Chanee Fabius, Stephanie Giordano, Eric Jutkowitz, Tetyana Shippee

**Affiliations:** University of Minnesota School of Public Health, Minneapolis, Minnesota, United States; Indiana University Bloomington, Bloomington, Indiana, United States; Johns Hopkins University, Baltimore, Maryland, United States; Human Services Research Institute, Cambridge, Massachusetts, United States; University of Minnesota, Minneapolis, Minnesota, United States; University of Minnesota, Minneapolis, Minnesota, United States

## Abstract

People living with physical disability (PD) receiving long-term services & supports (LTSS) experience greater unmet service needs. It is unclear if having a case manager for service coordination modifies PD-associated LTSS outcomes. To fill this knowledge gap, we evaluated associations of PD with emergency department (ED) visits and community integration among older LTSS consumers with and without a case manager. Among 6,756 respondents (age, ≥65 years) from the National Core Indicators- Aging & Disability Survey (2018-2019), we evaluated three dichotomized outcomes (yes vs no): ED visits (over 12 months); and self-reported community integration indicated by both- activity (i.e. being as active in community as preferred) and enjoyment (i.e. enjoying things outside home). We used logistic regression, adjusting for consumers’ sociodemographic and health-related characteristics, with random intercept for state. PD was documented in 60% of survey respondents. People living with PD were 27% more likely to report not having a case manager (p < 0.001). Among those without a case manager, PD was associated with significantly greater odds of ED visits (odds ratio [OR], 1.80, 95% CI, 1.48-2.19) and lower odds of community integration [activity: OR, 0.75, 95% CI, 0.62-0.90; enjoyment: OR, 0.69, 95% CI, 0.58-0.83]. These associations were attenuated among consumers with a case manager [ED visits: OR, 1.09, 95% CI, 0.94-1.25; activity: OR, 0.86, 95%CI, 0.75-1.00; enjoyment: OR, 0.85, 95% CI, 0.73-1.00]. Older LTSS consumers living with documented PD are more likely to report not having a case manager; and having a case manager might mitigate PD-associated adverse LTSS outcomes.

